# Harnessing the Genetic Basis of Sorghum Biomass-Related Traits to Facilitate Bioenergy Applications

**DOI:** 10.3390/ijms241914549

**Published:** 2023-09-26

**Authors:** Lin Yang, Qin Zhou, Xuan Sheng, Xiangqian Chen, Yuqing Hua, Shuang Lin, Qiyun Luo, Boju Yu, Ti Shao, Yixiao Wu, Junli Chang, Yin Li, Min Tu

**Affiliations:** 1 School of Chemical and Environmental Engineering, Wuhan Polytechnic University, Wuhan 430023, Chinawuyixiao@whpu.edu.cn (Y.W.); 2 School of Life Science and Technology, Wuhan Polytechnic University, Wuhan 430023, China; 3 The Genetic Engineering International Cooperation Base of Chinese Ministry of Science and Technology, The Key Laboratory of Molecular Biophysics of Chinese Ministry of Education, College of Life Science and Technology, Huazhong University of Science & Technology, Wuhan 430074, China; d202180784@hust.edu.cn (B.Y.); d202280827@mail.hust.edu.cn (T.S.); cjl@hust.edu.cn (J.C.)

**Keywords:** bioenergy crops, sorghum, biomass, bioenergy, biofuel, biomass-related traits, carbohydrate metabolism, internodes, candidate genes, quantitative trait loci

## Abstract

The extensive use of fossil fuels and global climate change have raised ever-increasing attention to sustainable development, global food security and the replacement of fossil fuels by renewable energy. Several C4 monocot grasses have excellent photosynthetic ability, stress tolerance and may rapidly produce biomass in marginal lands with low agronomic inputs, thus representing an important source of bioenergy. Among these grasses, *Sorghum bicolor* has been recognized as not only a promising bioenergy crop but also a research model due to its diploidy, simple genome, genetic diversity and clear orthologous relationship with other grass genomes, allowing sorghum research to be easily translated to other grasses. Although sorghum molecular genetic studies have lagged far behind those of major crops (e.g., rice and maize), recent advances have been made in a number of biomass-related traits to dissect the genetic loci and candidate genes, and to discover the functions of key genes. However, molecular and/or targeted breeding toward biomass-related traits in sorghum have not fully benefited from these pieces of genetic knowledge. Thus, to facilitate the breeding and bioenergy applications of sorghum, this perspective summarizes the bioenergy applications of different types of sorghum and outlines the genetic control of the biomass-related traits, ranging from flowering/maturity, plant height, internode morphological traits and metabolic compositions. In particular, we describe the dynamic changes of carbohydrate metabolism in sorghum internodes and highlight the molecular regulators involved in the different stages of internode carbohydrate metabolism, which affects the bioenergy utilization of sorghum biomass. We argue the way forward is to further enhance our understanding of the genetic mechanisms of these biomass-related traits with new technologies, which will lead to future directions toward tailored designing sorghum biomass traits suitable for different bioenergy applications.

## 1. Introduction

The wide use of fossil fuels, the dominant source of energy, has been playing a critical role in the rapid development of human society over the past century; however, the detrimental impacts have become apparent in recent years in numerous aspects of our daily life, ranging from environmental pollution, global warming and the unsustainability of energy use [1]. To alleviate the negative impacts and replace, at least partial, fossil fuels, increasing efforts and capitalization have been made on technologies and applications of various renewable energy, among which bioenergy produced from plant biomass represents an important bioenergy type. Plant species or varieties suitable for the production of biomass and bioenergy should have the following characteristics: (1) low inputs for cultivation including both water and nutrition requirements; (2) the rapid accumulation of biomass (such as dry matter or a particular type of biomass, i.e., lignocellulose or starch). Woody species (e.g., willow and poplar), aquatic plants (e.g., algae and duckweed) and many cereal grass species (e.g., sugarcane, switchgrass and *Miscanthus*) meet the above-mentioned characteristics and thus are considered as promising biomass plants. Wheat and rice straw and agricultural waste are produced in large amounts annually in China and India, serving as significant sources of biomass [2]. Both the upstream biological knowledge of biomass formation and downstream technologies for biomass/bioenergy utilizations are essential for promoting bioenergy applications. Bioenergy crops can be mainly classified into three generations: the first-generation bioenergy crops, which represent those food and/oil crops (e.g., corn, sugarcane, sorghum and rapeseed) that can be adapted to bioethanol or biodiesel production; the second-generation bioenergy crops, which stand for those grass species utilizing lignocellulosic biomass with higher environmental tolerance and lower cost of biofuel production, such as switchgrass, alfalfa and *Miscanthus*; and the third-generation bioenergy crops which include boreal plants, eucalyptus, microalgae and crassulacean acid metabolism (CAM) plants that are more compatible with cellulolytic bacteria for efficient fuel conversion [3].

This article is focused on the genetic control related to the biomass production of cereal grass species using *Sorghum bicolor* (L.) as a representative model. Sorghum ranks 5th in global cereal production. More importantly, sorghum plants hold several features to serve as the research model of other related bioenergy grasses: (1) several types of sorghum (e.g., sweet sorghum, forage sorghum and energy sorghum) have promising bioenergy utilizations; (2) sorghum has a relatively simple diploid genome in comparison to other bioenergy grass species, such as switchgrass, *Miscanthus* and sugarcane, whose genomes are complex, polyploidy and much larger in size; (3) sorghum, together with several other bioenergy grass species, has outstanding tolerance to many abiotic stresses (such as heat and drought), allowing the plants to grow to large amounts of biomass in barren or marginal lands but not to compete with staple crops for well-fertilized farm lands [4]. While the basic research of sorghum falls much behind the grass model species (i.e., rice and maize), numerous advances have still been made in the past decades owing to the importance of sorghum as a bioenergy crop and healthy grain cereal. Such advances have benefitted from not only molecular genetics and population genetics, but also the technological improvements and applications of genomics and multi-omics [5,6]. Many aspects of the recent achievements in sorghum have been reviewed, such as the genetic and genomic resources of sorghum [7], molecular and genomic breeding of sorghum [8], the genetic control of plant architecture traits of sorghum [9] and the improvements in sorghum transformation and gene editing [10]. Other review articles focused on particular sorghum types (i.e., the bioenergy attributes and techno-economic analysis of sweet sorghum and energy sorghum) are also valuable additions to the state-of-art of sorghum biology, highlighting the promising hotspots of sorghum research [2,11,12,13]. On one hand, bioconversion technologies have been established and improved to facilitate the production of biofuels from sorghum biomass or to generate value-added chemicals [14,15,16]; on the other hand, the effects of genetic modification and molecular breeding on biomass composition and yield remain largely unknown, as bioenergy and bioconversion applications usually used regular forage and/or sweet sorghum cultivars. Noteworthily, many genetic loci and important genes involved in the control of biomass-related traits have been recently uncovered by population genomics and molecular genetics approaches in sorghum. To facilitate the translation of sorghum genetic knowledge to bioenergy-purposed targeted breeding and improved bioenergy applications, we describe in this perspective the bioenergy application scenarios of sorghum, summarize the recent advances in the genes and loci regulating biomass-related traits and prospect the future direction of sorghum molecular breeding toward designing better bioenergy applications and efficiencies using the modern breeding technologies.

## 2. Bioenergy Scenarios of Sorghum

**Figure 1:**
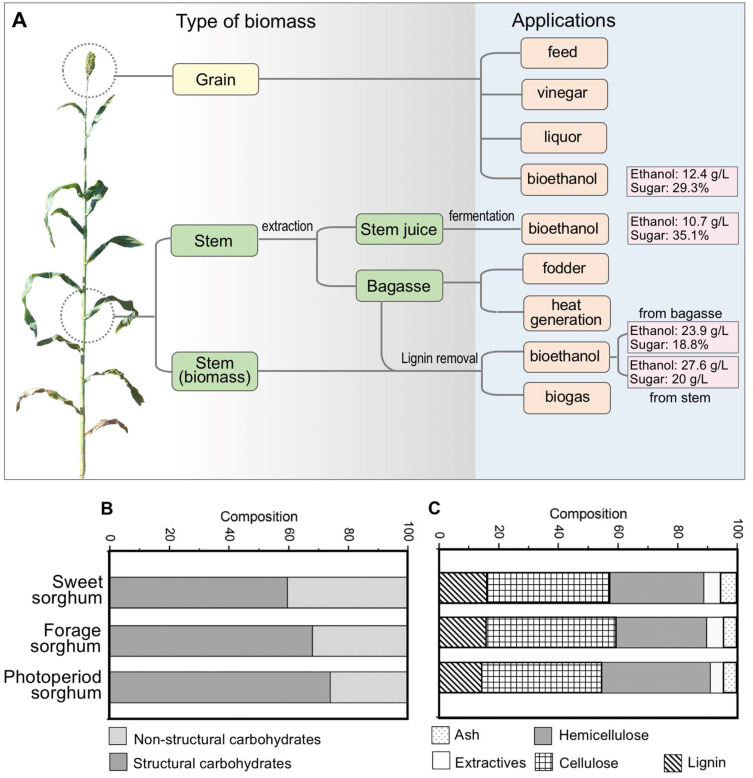
The types of sorghum biomass, bioenergy applications and compositions. (**A**) Sorghum plants produce different types of biomass (e.g., the grain, stem or stem bagasse), facilitating various bioenergy application scenarios. (**B**) Comparison of the percentages of structural and non-structural carbohydrates between sweet, forage and photoperiod sorghum. (**C**) Comparison of the constituents of lignocellulosic biomass between the sorghum types [32,33].

Different parts of sorghum plants can be harvested and dissected for distinct uses (Figure 1). In many countries, sorghum grains are used for food or feed, while the grains are also important materials for fermentation into liquor and vinegar [2,17]. The grain starch can be also directly fermented to produce biofuels, such as ethanol and butanol [16]. For sweet, forage and energy sorghum, their stalks are the main source of biomass with different percentages of non-structural (e.g., soluble sugars and starch) and structural carbohydrates and lignocellulosic carbohydrates (e.g., hemicellulose, cellulose and lignin) (Figure 1B,C). For example, sweet sorghum has a significantly higher proportion of non-structural carbohydrates, enabling its stems suitable for multi-purpose applications, such as biofuel production, heat generation and fodder usage (Figure 1A). Importantly, the bagasse (crushed residues of sorghum stalks after juice extraction) can usually be used for combustion to generate heat and electricity, which could serve as an additional energy source for rural areas in developing countries and regions (Figure 1A) [12,18]. The stalks of forage or energy sorghum plants are used for either biofuel or biogas production. Unlike biofuels, biogas is produced in aqueous environments through anaerobic digestion by decomposing the organic materials of sorghum stalks in the absence of air and presence of microorganisms [19]. The biogas generated from sorghum biomass mainly contains methane and carbon dioxide, ranging from 55% to 70% and 25% to 40%, respectively, with ammonia, hydrogen sulfide and hydrogen as the minor constituents. The major advantages of biogas production over biofuel conversion is that it has much lower technological requirements and thus is favored in developing nations and rural regions, helping to supply additional energy at low costs [20,21]. Factors affecting the utilization of sorghum biomass for biogas production has been studied and found that acidification and/or Organosolv pretreatments are valuable approaches to enhance biogas production [22,23].

Biofuels (typically ethanol and butanol) can be produced from either the dried or fresh sorghum stems, allowing versatile bioenergy workflow and scenarios. For dried stem-based biofuel production, enzymatic liquefication and/or hydrothermal pretreatments were found to help improve saccharification [24,25]. One major factor affecting the bioenergy utilization of sorghum is the biomass yield, which usually varies from approximately 15 to 25 Mg ha^−1^ for typical sweet sorghum cultivars. Such amounts of biomass could be translated to the theoretical ethanol yield ranging from 10,000 L ha^−1^ to 15,000 L ha^−1^ [13,26]. However, sorghum biomass yield could be dramatically increased through heterosis and/or breeding for bioenergy purposes with a yield potential to over 50 dry Mg ha^−1^ [11]. Biomass composition represents another key factor determining the suitable bioconversion path, which is largely affected by the stem juiciness trait. Many sorghum cultivars have juicy stems with relatively high water contents in the stem (>50%), fitting for separate utilizations of the extracted juice and bagasse (the resultant waste material after juice extraction). Sweet sorghum cultivars with a large amount of biomass are particularly suitable for the above-mentioned bioenergy utilization, as the composition of the sweet sorghum stem includes juice (averagely ~73%), soluble sugars (>13%), cellulose (5.3%), hemicellulose (3.7%) and lignin (2.7%) [27]. The extracted stem juice can be directly fermented to biofuel, while the bagasse can be further subject to lignin removal and biofuel or biogas generation [28]. The bagasse can be used as fodder, too. Pretreatments by physical, chemical or biological methods are required to remove lignin and then hydrolyzed by enzymes (cellulase and hemi-cellulase) to convert holocellulose to fermentable sugars, which could be further produced to ethanol, butanol and other chemicals (e.g., acetone, acetate, butyrate) or value-added bio-products using different microorganisms [16,29,30,31,32,33].

## 3. Genetic Control of the Biomass-Related Traits in Sorghum

**Figure 2:**
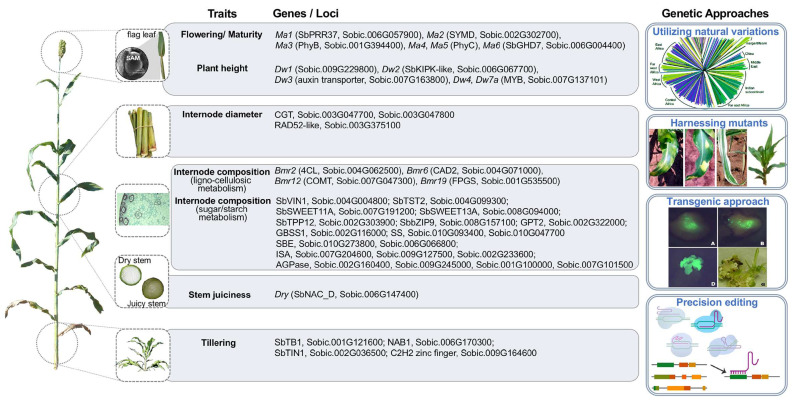
A diagram to summarize the biomass-related traits in sorghum, the current knowledge regarding the genetic control of these traits and possible genetic approaches to enhance the biomass traits. The traits are categorized into several groups: plant height, flowering/maturity, internode morphology (i.e., internode number, internode length, internode diameter), internode composition (including both lignocellulosic metabolism and sugar/starch metabolism), stem juiciness, and tillering. These genes have been previously reported to regulate or be associated with these traits. Sorghum varieties with excellent bioenergy performance could be accurately designed by pyramiding the superior alleles of important genes and genetic loci.

For tailored designing sorghum plants with bioenergy purposes, the genetic control of biomass-related traits, including major quantitative genetic loci (QTL) and key genes, are the prerequisite. This section presents the current knowledge of the molecular genetic basis of sorghum biomass traits (Figure 2), which ranges from developmental traits (e.g., flowering and maturity), plant architecture traits (e.g., plant height, tiller number), and metabolic traits (such as those related to lignocellulose, sugar or starch metabolism).

### 3.1. Tillering

The manipulation of tillering could optimize plant architecture and ultimately contribute to biomass and grain yield in sorghum [34], as the tiller number affects both the growth of the main stalk and tillers. However, tillering is a complex trait that is involved in the interplay between phytohormones and nutrient signaling and is affected by both genotype and environmental factors [35]. Previous studies have shown that the sorghum orthologue (Sobic.001G121600) of the maize classical domestication gene *tb1*, which encodes a basic helix–loop–helix transcription factor, regulates the tiller number in sorghum, and its expression is affected by phytochrome B (phyB) [36]. Studies using sorghum mutants also shed light on the genetic control of tillering. Functional characterization and positional cloning of the causal gene of a *non-dormant axillary bud 1* (*nab1*) mutant identified the *NAB1* gene (Sobic.006G170300) encoding a carotenoid cleavage dioxygenase, which is thought to be involved in the biosynthesis of gibberellin lactones [37]. *NAB1* is predominantly expressed at axillary bud nodes and tiller bases to reduce the till number and the nab1 mutant shows an increased tiller number but decreased plant height. In addition, several high tilling mutants were also found in the gamma-ray induced mutant pool, suggesting that till number is a useful trait that could still be improved to optimize the whole-plant biomass of sorghum [38]. GWAS analysis in sorghum identifies Sobic.009G164600, encoding a C2H2 zinc finger protein, as a candidate gene for tiller number regulation. In maize, *tin1*, also encoding a C2H2 zinc finger protein, has been demonstrated to control the tiller number with conserved functions across cereal crops [39,40]. The study of maize *tin1* suggests the sorghum orthologous gene, Sobic.002G036500, might also play a role in tiller number regulation, deserving further functional validation.

### 3.2. Plant Height

Plant height is generally considered to be proportional to biomass yield. Up to now, genetic and molecular breeding evidence strongly suggests that the biomass of sorghum plants could be enhanced through optimizing plant height. Five major genetic loci have been known to control internode length and hence the plant height of sorghum: *Dw1*, *Dw2*, *Dw3*, *Dw4* and *Dw7a* (‘*Dw*’ indicates ‘dwarfism’) [40,41]. *Dw1* encodes a membrane protein of unknown function that is highly conserved in plants [42] and *Dw1* reduces the proliferative activity of stem internode cells [43]. A recent functional study found that DW1 interacted with a negative regulator of brassinosteroid signaling, BRASSINOSTEROID INSENSITIVE 2 (BIN2) and inhibited BIN2 function by preventing its importation to nuclear [44]. *Dw2* encodes a protein kinase homologous to the AGCV III protein kinase KIPK [45]. The *Dw2* alleles have been used extensively in U.S. grain sorghum breeding programs and sorghum conversion programs to reduce the stem length in sorghum genotypes (e.g., IS3620c and BTx642). *Dw3*, as the first dwarfism gene cloned in sorghum, is considered to be the most important locus controlling differences in plant height between the tall and short sorghum genotypes [46,47], which is orthologous to the maize *brachytic2* (*br2*) gene encoding an ABCB1 auxin efflux transporter. *Dw3* also contributes to improved lodging resistance [43]. More recently, *Dw7a* has been discovered as a new locus associated with plant height, with a candidate gene encoding an R2R3-type MYB transcription factor [41]. The recessive allele of *Dw7a* confers reduced internode length and decreased plant height. Comprehensive population genetics studies revealed other genetic loci associated with sorghum plant height [48,49,50], while the causal genes for many of these loci (such as *Dw4* and *qHT7.1*) have not been cloned and functionally characterized. The way forward is to identify the genes that control sorghum plant height, to uncover the underlying molecular mechanisms and to establish the genotype–phenotype relationships for these genes and their alleles in diverse sets of sorghum germplasm.

### 3.3. Flowering/Maturity

Flowering is one of the key events of plant development and the flowering time marks the end of the asexual reproduction phase. In cereal crops, delayed flowering could usually lead to a longer growth period and increased biomass accumulation. Sorghum is a short-day tropical graminaceous plant and many sorghum accessions are sensitive to photoperiod. Therefore, the photoperiod-dependent flowering (known as “maturity”) represents one of the key agronomic traits in sorghum breeding. For the photoperiod sensitive sorghum genotypes (also known as photoperiod sorghum, Figure 1), flowering occurs later or not at all under long days compared to short days, while photoperiod-insensitive sorghum genotypes do not respond to the photoperiod to initiate the flowering event [11,51].

Extensive genetic analyses have established the genetic architecture of maturity in sorghum, controlled by the *Ma1* to *Ma6* loci [36,52,53]. These loci control photoperiod-dependent flowering in sorghum, and consequently, possibly affect biomass accumulation.

*Ma1* encodes the flowering repressor, sorghum Pseudo-Response Regulator 37 (SbPRR37), a component of the circadian clock controlling system conserved in multiple plant species, which is regulated by circadian rhythms and inhibits flowering during long days [54]. *Ma3* (Sobic.001G394400) encodes phytochrome B (PhyB), a red-light photoreceptor that plays an important role in photoperiodic sensing. Upon sensing light signals, Ma3 represses the expression of the *tb1*(*Teosinte Branched 1*) gene and *DRM1* (dormancy-related gene), leading to axillary bud growth [35,55]. Similarly, *Ma2*, *Ma4*, *Ma5*, and *Ma6* have been associated with photoperiod sensitivity in sorghum [53,56,57,58,59]. Two loci, *Ma5* and *Ma6*, have been reported to enhance photoperiodic sensitivity and prolong the asexual growth period [58,60]. Recently, *Ma6* has been cloned and characterized [60]. *Ma6* encodes SbGhd7 and functions as a repressor of Early Hibernation Day 1 (SbEHD1) which represses flowering in sorghum during long day light [60]. The dominant alleles of these *Maturity* loci, respectively, contribute to delayed flowering in sorghum under long day conditions [60]. Among them, *Ma1* exhibits the greatest effect on photoperiod-dependent flowering [61]. While the molecular mechanisms of maturity and the genetic interactions between these *Ma* loci have not been fully revealed, these loci, at least some loci and some alleles, have been widely used in sorghum breeding programs. By selecting for the recessive alleles of *Ma1*, plant breeders in the United States have developed early flowering sorghum cultivars to ensure adequate time to grain maturity and to avoid frost damage, thereby increasing grain yields in temperate regions [62].

### 3.4. Stem Diameter

Sorghum stem diameter is a very important agronomic trait, which is positively associated with biomass as well as internode strength and lodging resistance. Recently, a study combining GWAS and RNA-seq identified two loci associated with the stem diameter, with one locus containing two duplicated genes (Sobic.003G047700 and Sobic.003G047800), both encoding the cytokinin biosynthetic enzyme, cytokinin-O-glucosyltransferase 3, and the other candidate gene (Sobic.003G375100) encoding mitochondrial DNA repair RAD52-like protein 1 [63]. Other studies using RIL populations identified many QTL regions for stem diameter located across almost all chromosomes in the sorghum genome [64,65]. In addition, thick-stem sorghum mutants have been identified in a gamma-ray-induced mutant library, providing a valuable genetic resource for elucidating the genetic factors controlling stem diameter [38].

### 3.5. Internode Juiciness

**Figure 3:**
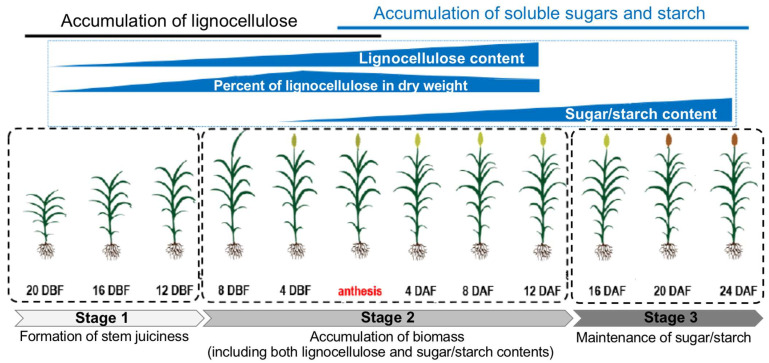
The dynamic process of stem elongation, biomass accumulation and changes in internode composition in sweet sorghum. The stem elongation and biomass accumulation process could be separated into three stages: **Stage 1**, around the end of stem elongation, programmed cell death is not initiated in sweet sorghum to keep internode parenchyma cells alive, forming the juicy stem, which is the basis of soluble sugar and/or starch accumulation in the stem; **Stage 2**, After stem elongation and before anthesis, soluble sugars are rapidly increased in the stems of sweet sorghum cultivars, reaching up to ~23% of the extracted stem juice; **Stage 3**, During the late stages of grain development, the starch content in the internode rises up, reaching up to ~10% of the stem dry weight. DAF, days after flowering; DBF, days before flowering. Anthesis stage is highlighted in red.

In addition to the above-mentioned developmental and morphological traits, metabolic diversity has been observed among different sorghum types and genotypes [66,67] and affects the composition of the sorghum stem and the corresponding bioenergy utilization. Sorghum stems can be generally divided into two types: dried stem and juicy stem (Figure 2) [68], which has long been known to be controlled by a classical genetic locus, the *Dry/D* locus [69,70]. During sorghum stem development, internode cells are regulated by programmed cell death (PCD) and become dried, forming the dried-stem phenotype and leaving lignocellulosic biomass, while in some other genotypes (e.g., many sweet sorghum cultivars), internode cells do not experience the PCD process after stem elongation, producing the juicy-stem phenotype, and could accumulate a large amount of soluble sugars (mainly sucrose, glucose and fructose) around the flowering time and even a considerable amount of starch during later stages [71,72,73]. Taken together, the dynamic process of stem juiciness and metabolic changes can be separated into three stages (Figure 3): **Stage 1** represents the formation of stem juiciness, which could decide the bioenergy utilization of the sorghum stem biomass (using lignocellulosic biomass to convert to biofuels or first extracting stem juice for biofuel production); **Stage 2** is the stage when soluble sugar is rapidly accumulated in stem parenchyma cells, which could determine the quality of sorghum stem juice for direct fermentation to biofuels; **Stage 3** represents a period of time when parts of the soluble sugar are converted to starch in the stem and the content of soluble sugar could be decreased, likely due to genetic and/or environmental factors (e.g., low temperature) [73,74]. Thus, **Stage 3** affects the length of the period suitable for harvesting sorghum biomass. The known candidate genes determining stem juiciness, lignocellulose and sugar/starch metabolism, respectively, in the sorghum stem are summarized as follows based on the three stages.

Recently, the causal gene of the *Dry* locus has been cloned (Sobic.006G147400), encoding a NAC transcription factor (TF), which was proposed as a master regulator of PCD during sorghum stem development. In the dried-stem sorghum genotypes, the *Dry* allele encodes a functional NAC TF (namely *SbNAC_D*) to cause parenchyma cell death after stem elongation, while the detailed molecular mechanisms for *SbNAC_D*-induced PCD remain to be characterized. By contrast, in the juicy-stem sorghum genotypes, loss-of-function *dry* alleles could not induce PCD, leaving stem parenchyma cells alive and capable for sugar accumulation [65,71,75,76].

### 3.6. Internode Cell Wall Metabolism

During the stem elongation and post-elongation processes, both of the biosyntheses of the primary and secondary cell wall are involved. These processes include the metabolism of cellulose, hemicellulose, phenyl-propanoids and mono-lignols, as well as cell wall reassembly and degradation. In sorghum, p-hydroxyphenyl (H), guaiacyl (G) and butyryl (S) monolignols are the main monolignols synthesized via the phenylpropanoid pathway [77]. Time-series RNA-seq analyses of sorghum internodes and mutant analyses contribute to the identification of important candidate genes influencing internode cell wall composition. Subsequently, cell wall composition or the lignocellulosic composition of sorghum stems is one of the critical factors affecting the sorghum bioenergy utilization scenario and efficiency. On one hand, a comprehensive genome-wide identification has discovered 520 genes from 20 gene families related to biosynthesis and/or the modification of various cell wall polymers (e.g., cellulose, hemicellulose, pectin, and lignin) [78]. More recently, a number of MYB and NAC TF-encoding genes have been identified in sorghum as the orthologs of those in Arabidopsis and other species, which are known to be important regulators for secondary cell wall accumulation [79]. These large-scale gene identification analyses serve as the starting point for pinpointing the key candidate genes affecting sorghum stem composition and bioenergy applications. On the other hand, forward genetic studies in sorghum have revealed the link between the *brown midrib* (*bmr*) phenotype and lignocellulose conversion (particularly, reduced lignin contents and increased saccharification rate). Owing to the connections with bioenergy applications, *bmr* mutants have been extensively screened in various sorghum populations to identify the genes/loci that could reduce the lignin content and/or modify biomass composition, thereby increasing sorghum biomass conversion [80,81,82,83]. *Bmr-2* mutants result in decreased G-units and S-units [80,83]; *bmr-6* mutants lead to lowered lignin contents, a reduced number of monolignol G-units and an increase in cinnamaldehyde content [84], whereas *bmr-12* alleles caused lowered lignin contents and a positive effect on lignocellulose conversion and digestion efficiency [85]. Mutations of *bmr-19* are associated with significantly reduced lignin contents and the candidate gene underlying this *bmr* mutation has been identified as a putative *folylpolyglutamate synthase* (*FPGS*) gene (Sobic.001G535500) [80,82]. Among these four *bmr* alleles, *bmr-6* and *bmr-12* have been widely used in sorghum breeding programs. The causal genes for *Bmr-2, Bmr-6* and *Bmr-12* have been cloned, encoding 4-coumarate coenzyme A ligase (4CL, Sobic.004G062500), cinnamyl alcohol dehydrogenase 2 (CAD2, Sobic.004G071000) and caffeic acid O-methyltransferase (COMT, Sobic.007G047300), respectively [83,86,87].

RNA-seq, together with other omics technologies, has been making a huge contribution to pinpointing the key genes associated with cell wall metabolism and biomass accumulation in the sorghum stem. Time-series transcriptome of the sweet sorghum cultivar Della revealed the concerted down-regulation of genes involved in the synthesis of cellulose, hemicellulose, lignin, glucurono-arabinoxylan and expansins, with a clear transition time point around flowering [88]. Another detailed comparative transcriptome analysis of three sorghum genotypes (Rio, R9188 and BTx406) revealed the differences of cell-wall metabolic gene expression between sugar-accumulating sorghum stems and non-sugar-accumulating stems that are biosynthetic genes for cellulose; cellulose-like, pectin and some glycoside hydrolase-encoding genes had higher expression in the sweet sorghum stems [73]. The comparison between different sorghum genotypes and the comparison between stem regions within the same genotypes provide new insights into the stem vegetative growth and intercalary meristem development [89,90]. A detailed analysis of the sub-apical internodes (in bioenergy sorghum R.07020) indicated that internode growth involves the up-regulation of auxin signaling and the metabolism and signaling of other phytohormones (i.e., GA, BR, CK, ABA and JA) [89]. A more recent study focused on the transcriptomes of distinct regions within the internode localized the intercalary meristem (IM) and provided insights into how GA and IAA signaling and the downstream gene networks regulate cell proliferation in sorghum stems [90]. During the post-elongation stages, comparative RNA-seq analysis indicated that cell wall metabolism appeared to be more active in the sweet sorghum genotypes than the other genotypes low in biomass and stem sugar. For example, some *CesA* genes encoding the cellulose synthase for primary cell wall were highly expressed in sweet sorghum lines, and many mono-lignol biosynthetic genes (e.g., *PAL*, *C4H*, *4CL*, *HCT*, *CCR*, *CAD*, *COMT* and *CCoAOMT*) were down-regulated slower in the sweet sorghum lines than the other sorghum genotypes [91]. It is worth highlighting that RNA-seq analysis has played a critical role in identifying the key candidate genes associated with several biological events of sorghum biomass accumulation, such as internode growth, elongation, cell wall biosynthesis and sugar and starch accumulation, as the high expression levels of particular members within a given gene family and the correlation between time-course gene expression data and the accumulation profiles of a biomass component or metabolite are important, valuable information for gene identification in sorghum.

### 3.7. Internode Sugar and Starch Metabolism

The metabolic analysis of sorghum internodes and stem juice demonstrated that sucrose, glucose and fructose are the main non-structural carbohydrates with sucrose being the most dominant soluble sugar in most sweet sorghum genotypes [67,92]. RNA-seq also contributes to the identification of potential sugar transporters and regulatory proteins involved in stem sugar accumulation. Several analyses consistently showed that the down-regulation of vacuolar invertase (*SbVIN1*, Sobic.004G004800) likely serves as the prerequisite for sugar accumulation in the vacuoles of stem parenchyma cells and the up-regulation of tonoplast sugar transporter (*SbTST2*, Sobic.004G099300) may probably be the major transporter responsible for the uptake of sugar into vacuoles [73,88,91,93]. The roles of other sugar transporters in sorghum stem sugar accumulation remains to be investigated. Among the six sorghum sucrose transporters (SbSUT), *SbSUT1* (Sobic.001G488700), *SbSUT4* (Sobic.008G193300), and *SbSUT6* (Sobic.007G214500) were highly expressed in leaf tissues while *SbSUT2* (Sobic.004G353600) and *SbSUT5* (Sobic.004G190500) were expressed in stem tissues, suggesting their distinct roles in the source and sink [94,95]. Twenty-three *Sugar Will Eventually be Exported Transporters* (*SWEETs*) were identified, falling into four phylogenetically conserved clades [91], and *SbSWEET11A* and *SbSWEET13A* (Sobic.007G191200 and Sobic.008G094000, respectively) might be involved in sugar exportation during the stem sugar accumulation process [91,96]. Despite these above-mentioned advances in sorghum sugar transporters, our knowledge about the Stage 3 of stem sugar accumulation is relatively scarce. By leveraging genetics, transcriptomics and metabolomics, Li et al. revealed the important role of the sucrose-signaling trehalose-6-phosphate (T6P) pathway in maintaining high sugar contents, and identified the candidate genes potentially regulating this process (*SbTPP12*, Sobic.002G303900, *SbbZIP9*, Sobic.008G157100) [73]. These results suggest that sugar accumulation is a dynamic process in the sorghum stem and the maintenance of high sugar contents requires T6P signaling-mediated regulatory networks possibly involved in multiple carbohydrate storage and utilization pathways. In general, a systematic view and orchestration among the sugar transporters during the sorghum stem sugar accumulation process remains incomplete, because: (1) the major facilitator superfamily (MFS) includes many other sub-families that could transport sucrose, glucose or fructose but have not been characterized in sweet and bioenergy sorghum genotypes, such as the sugar transporter family (STP), hexose transporter family (HT), plastidic glucose transporter family (pGlcT), vacuolar glucose transporter family (VGT), inositol transporter family (INT), polyol transporter family (PLT) and the sugar facilitator protein family (SFP) [97,98]; (2) pan-genomic or population genomic investigations are still lacking in sorghum for the numerous transporter-encoding genes to establish the gene-phenotype correlations [99,100].

In addition to the soluble sugars accumulated in the stem, starch represents another source of biomass in the sorghum stem that could be readily fitted to bioenergy applications. Importantly, starch appears to be accumulated at a higher percentage in sweet sorghum than in grain sorghum (3%~9% in sweet sorghum genotypes versus less than 1% in grain sorghum genotypes) [72]. Starch was accumulated in the same sets of internodes where sugar was highly accumulated. RNA-seq studies systematically portraited the expression dynamics of starch metabolic genes during stem developmental stages and identified a number of highly expressed starch biosynthetic genes that showed coordinated up-regulation during the late stages of sugar accumulation, including glucose-6-phosphate translocator (*GPT2*, Sobic.002G322000), *AGPase* (Sobic.002G160400, Sobic.009G245000, Sobic.001G100000, Sobic.007G101500), starch synthase (*SS*, Sobic.010G093400, Sobic.010G047700), isoamylase (*ISA*, Sobic.007G204600, Sobic.009G127500, Sobic.002G233600), granule bound starch synthase (*GBSS1*, Sobic.002G116000) and starch branching enzyme (*SBE*, Sobic.010G273800, Sobic.006G066800) [72,91]. These genes are important candidates for manipulating the starch accumulation and carbohydrate composition in sorghum stems.

Other approaches have also identified candidate genes associated with stem sugar accumulation in sorghum. For instance, genome-wide association analysis (GWAS) identified tandem duplicated vacuolar iron transporters (*VIT1*, Sobic.004G301500 and Sobic.004G301600) as candidate genes for the difference in stem water-soluble carbohydrate in the sorghum population [101]. MicroRNA (miRNA) sequencing studies identified several miRNAs (such as miR169, miR164 and miR166) that were either differentially expressed between sweet and grain sorghum genotypes or sugar-accumulating stages, suggesting their potential involvement in stem sugar accumulation and/or carbohydrate allocation in sorghum [102,103,104,105]. These candidate genes and miRNAs provide addition clues to enrich the complex molecular networks controlling carbohydrate allocation in the sorghum stem and deserve further investigations.

## 4. Challenges Associated with Utilizing the Biomass-Related Genetic Knowledge

While the genetic knowledge regarding biomass-related traits has been greatly enhanced, several challenges remain to be resolved before biomass yield and composition could be readily designed in sorghum. First, to what extent genetic control can help to increase biomass or to optimize biomass composition still needs more investigations. Owing to the technical challenges of generating transgenic or gene-edited sorghum plants, the effects on biomass yield or composition were only characterized in detail for a few genes/loci. For example, in sudangrass (*Sorghum bicolor* subsp. *drummondii* (Nees ex Steud.)), field trial results demonstrated that the *bmr* lines averaged 9% lower in lignin and 7.2% higher in in vitro fiber digestibility than normal lines [106]. By comparison, sorghum *brown midrib* mutants are generally considered to be associated with lower biomass yields. Previous yield trials reported an average of 12% yield loss for the *bmr* sorghum hybrids [107,108,109]. On the other hand, *bmr* mutations indeed brought clear benefits to better utilize the lignocellulosic biomass of sorghum. For instance, the *bmr-6*, *bmr-12* single mutants and *bmr-6/12* double mutant reduced lignin content by 13%, 15% and 27%, respectively, while increasing glucose yields for the sorghum biomass by 27%, 23% and 34%, respectively. With dilute-acid pretreatment and simultaneous saccharification and fermentation by cellulases and *Saccharomyces cerevisiae*, bioethanol conversion was improved by 22%, 21% and 43% for *bmr-6*, *bmr-12* and *bmr-6/12* mutants, respectively [110]. The genetic effects of *SbNAC_D* and *soluble acid invertase 2* (*SbSAI2*, the ortholog of rice *VIN2*) were also known. Expression of the functional *SbNAC_D* allele in the sweet sorghum background (cv. Keller) led to a significant drop of stem water content from over 70% to 60% [78]. In the rice plants overexpressing SbSAI2, the contents of glucose and fructose were increased by two- and three-fold, respectively, with the sucrose content decreased to undetectable [111].

The second challenge is that genetic transformation and gene editing of sorghum have not become routine techniques for the sorghum community with a number of limitations such as lower transformation efficiencies compared with those of maize and rice, and the genotype limitation. We will not expand to the details of recent advances of sorghum transformation as it has been well reviewed [10]. In brief, sorghum can be transformed with particle bombardment- and *Agrobacterium*-based methods, with transformation efficiencies varying from <1% to a maximum of 46%. While recent improvements in tissue culture procedures have allowed a higher transformation efficiency (from 8.3% to 36%), the technique is still not widely applied in many laboratories working on sorghum (reviewed by Silva [10]). Moreover, the genotypes and explant forms suitable for sorghum transformation are very limited. In addition, gene editing using the CRISPR/Cas-mediated method has been established in sorghum [112,113,114]. Further improvement on sorghum transformation and gene editing will definitely pave the way toward understanding the effects of genetic manipulation on biomass yield and composition. More importantly, these technologies are critical components of molecular breeding, providing a fast route to generate new sorghum germplasm with desirable biomass-related phenotypes (Figure 2).

The third challenge for the improvement of sorghum biomass and bioenergy traits is likely the complex genetic interactions between the genes/loci and the effects of genetic backgrounds. Genetic interactions between different genes and loci that influence the same set of traits (lignocellulosic composition, for instance) are still not well characterized, hampering the precise designing of the target traits with gene pyramiding. The recent pan-genomic study of sorghum revealed that 63.6% of the genes are shell genes that are only present in some genotypes but absent in the others [108]. Extensive genetic variations among sorghum genotypes and races could be a challenge when translating the knowledge from the commonly used genotypes (i.e., BTx623, Tx430, Rio) to those widely planted.

## 5. Concluding Remarks

In summary, recent advances in the identified sorghum genetic loci/genes regulating biomass-related traits will allow molecular breeding possible to design the biomass amount and composition of sorghum plants to better facilitate bioenergy applications. It has to be acknowledged that the current understanding of the genetic control of sorghum biomass-related traits is just the starting point but far from sufficient for accurately tailoring the biomass traits. Many genetic loci could be now identified through GWAS analysis in sorghum; however, most of the causal genes are yet to be discovered [115,116,117]. Recent technological breakthroughs should be harnessed to further gene functional studies in bioenergy sorghum. First, the efficiency of sorghum transformation has to be improved and efficient gene editing has to be achieved in sorghum. Further optimization and the wide applications of these techniques throughout the sorghum community is critical to identify the causal genes and uncover the underlying molecular mechanisms. Second, single-cell transcriptome and spatial transcriptome and metabolome have been successfully applied and provided novel insights into numerous biological questions in various plant species, especially those of which transformation remains challenging [118,119,120,121,122]. These advanced omics technologies are believed to further boost our gene identification and mechanistic analyses toward the biomass-related traits. Third, establishing the genotype–phenotype relationships and discovering superior alleles or haplotypes for bioenergy improvement across diverse sorghum populations will facilitate molecular breeding and genomic selection in sorghum. The new pan-genomic resources and integrated sorghum multi-omics databases (e.g., SorghumBase, Sorghum Genome Science Database (SorGSD) and MOROKOSHI) marked sorghum research entering into a new stage during the post-genomic era [100,123,124,125]. By utilizing and integrating these technologies and resources, we believe that our understanding of the genetic control of sorghum biomass-related traits will be greatly improved and will ultimately bring molecular design breeding for sorghum bioenergy applications.

## Data Availability

Data are contained within the article.
